# Self-Help Seeking for People Concerned About Their Thoughts and Behaviors Regarding Children During the COVID-19 Pandemic

**DOI:** 10.5964/sotrap.14301

**Published:** 2025-06-12

**Authors:** Michael C. Seto, Kailey Roche, Jenny Coleman, Donald Findlater, Elizabeth J. Letourneau

**Affiliations:** 1 Royal Ottawa Health Care Group, Ottawa, Ontario, Canada; 2 Stop It Now! USA, Northampton, MA, USA; 3 Lucy Faithfull Foundation, Epsom, United Kingdom; 4 Stop It Now! UK and Ireland, Epsom, United Kingdom; 5 Johns Hopkins University, Baltimore, MD, USA; University Medical Center Mainz, Mainz, Germany

**Keywords:** child sexual abuse, prevention, self-help, COVID-19

## Abstract

Concerns about the impacts of the COVID-19 pandemic on child sexual exploitation and abuse have been expressed by police and child protection organizations. However, there are limited data about how the pandemic may have caused changes in the help-seeking behavior of people who have concerns about their thoughts and behaviors regarding children. In this study, we examine helpline and webpage metrics from two organizations – Stop It Now! USA and Lucy Faithfull Foundation’s Stop It Now! UK and Ireland – providing resources about child sexual exploitation and abuse prevention, including self-help pages for people concerned about their sexual thoughts or behaviors involving children. Pages for self-help seekers were compared to pages for parents/caregivers and general audiences. Based on descriptive data, there was mixed evidence of an increased demand for self-help, with an increase in helpline inquiries and an increase in views for some but not all self-help pages. Webpage trends for self-help pages were not matched by similar trends for parent/caregiver or general information pages, suggesting the increase in demand was specific to self-help seeking. However, ANOVAs of both US and UK and Ireland webpage data did not result in a significant interaction effect between category (self-help, parent, or general) and time. While there were significant increases in self-help related helpline calls in the USA across time, all three types of helpline calls increased in the UK and Ireland. The implications of these data for self-help resources and perpetration prevention are discussed.

## Highlights

- Helplines and self-help resources are used by individuals concerned about their sexual thoughts or feelings about children.
- Engagement with helplines and self-help resources can be affected by context, including media awareness campaigns and major events like pandemic restrictions.
- Self-help resources for concerns about related children is a major gap in perpetration prevention.

## Background

Early in the pandemic, there were dire predictions of the negative impact of pandemic restrictions on the safety of children. Concern about increased child physical abuse centered on children being stuck at home with parents under stressful circumstances where abuse was less likely to be reported to or detected by teachers or other adults (Thompson, 2020). There were also concerns that children engaged in remote learning might be at greater risk of online child sexual exploitation or abuse, including sexual solicitations from adults (United Nations Children’s Fund [UNICEF], 2020). In addition, there were concerns that individuals at risk of perpetrating child sexual exploitation or abuse might be at greater risk because of stress, lack of positive social supports, barriers to help-seeking, and increased opportunity (e.g., being online more in the privacy of their own homes), all of which are associated with risk of offending (see Babchishin et al., 2018; Seto, 2013). Models of child maltreatment would predict an increase in some forms of maltreatment as a result of more stress, social isolation, and increased opportunity within the home (more time together) (e.g., stress and coping model, see Hillson & Kuiper, 1994).

Initial data about child sexual exploitation and abuse suggested increased police complaints and demand for child protection and social services during the pandemic versus pre-pandemic (e.g., INTERPOL, 2020; National Center for Missing and Exploited Children [NCMEC], 2021). For example, the NCMEC reported twice as many online enticement (solicitation) reports for 2020 compared to 2019, while the number of reports regarding child sexual exploitation materials (CSEM) increased by 63% across this same time period. The Royal Canadian Mounted Police (RCMP) reported a 36% increase in reports to their National Child Exploitation Crime Centre from March 2020 to May 2020, with a decline back to typical levels in June 2020, corresponding to the first major lockdown in Canada (Schlosser & Estey, 2021). The RCMP also reported an increase in darknet^1^1The darknet (or darkweb) is an unindexed portion of the internet which requires special software to access hidden sites using addresses built on randomly generated keys. The darknet can be used to anonymously access “clearnet” sites, but has often been connected to criminal activities, such as accessing child sexual exploitation material (Owen & Savage, 2015). chat, including conversations about taking advantage of specific features of the pandemic that might increase access to or vulnerability of children, such as social media platform vulnerabilities. Preliminary data from McMahan et al. (2020) also indicated more users on darknet forums where CSEM were shared. For example, on one of the largest darknet forums, there was an increase from 150 pre-pandemic to 400 pandemic active users at one time. Of particular concern, McMahan et al. (2020) also found that the discussions within the forums were veering more toward opportunities to offend given stay-at-home orders, as well as instructions on how to access children to produce CSEM. Indeed, in a qualitative study on the self-reported effects of the COVID-19 pandemic on help-seeking individuals accessing CSEM, McMahan et al. (2024) reported that participants endorsed increased urges or temptations to use CSEM due to stress as well as more time at home online. Further, in 2022, INTERPOL released a statement that online child sexual abuse has been increasing at a consistent rate since 2020.

Other analyses suggest there was no change or even a decline in child maltreatment reporting – including child sexual abuse reporting – early in the pandemic (Rapoport et al., 2021; Sharma et al., 2021). This was attributed to disruptions in child protection services; a UNICEF (2020) report found pandemic-related disruptions in child protective services were reported in 104 countries representing 1.8 billion children, excluding North America, the United Kingdom, the European Union, and Australia and New Zealand. Australia noted a decrease in reports of child maltreatment during the first phase of the pandemic, and then a rebound as restrictions eased (Australian Institute of Health and Welfare, 2021).

## Self-Help-Seeking for Sexual Attraction to Children

In general, people reported increased mental health difficulties during the COVID-19 pandemic lockdowns (Upton et al., 2023). In the United States, use of mental health resources increased by 18% in areas of the United States with lockdown restrictions compared to 1% in areas without restrictions (Ferwana & Varshney, 2024). Due to a reduction of in-person services, many individuals experiencing mental health difficulties turned to online resources to meet their mental health needs. Internet search engines, such as Google, were the most common method of help-seeking (Yonemoto & Kawashima, 2023).

Even prior to COVID-19 restrictions, people concerned with their sexual thoughts and behaviors related to children sought help online and through helplines as it affords a higher degree of privacy and anonymity. Privacy and anonymity may be especially important for individuals who are attracted to children due to the stigma associated with this attraction (Jahnke, 2018). Indeed, fear of negative judgement and being reported to the authorities are common barriers to help-seeking in people who are attracted to children (Grady et al., 2019).

An early adopter of the helpline model for providing prevention services to people concerned about their sexual thoughts and behaviors related to children is Stop It Now! Developed in the United States in 1992, the original goal of Stop It Now! was to involve adults, families, and communities in the protection of children and the prevention of child sexual abuse. In 2002, Stop It Now! expanded to the United Kingdom (Stop It Now! UK and Ireland). The Stop It Now! websites provide information about child sexual abuse prevention for families, children/youth, individuals who are concerned about their own thoughts/behaviors involving children, and professionals. The organization also provides two hotlines which people can contact anonymously to express their concerns or gather more information. These hotlines are both phone-based and chat-based, allowing for ease of access.

From the increased reporting of child sexual exploitation, as well as an increase in general mental health difficulties during COVID lockdowns, it follows that individuals concerned about their sexual thoughts and behaviors related to children would also have the need to reach out to help lines and websites for services. To this end, we examined whether there was an increase in Stop It Now! website traffic and helpline contact during COVID-19. Analyzing website and helpline traffic can not only indicate whether there was an increase in people seeking out help for their sexual thoughts and behaviors but might also inform us as to what types of information people are seeking and provide justification for building up programs for the prevention of child sexual abuse and exploitation. While other researchers have examined general mental health helpline metrics and caller behavior across similar time periods before and during COVID-19 restrictions (e.g., Scerri et al., 2021; Turkington et al., 2020), to our knowledge, there has been no analysis of website and helpline metrics for programs related to preventing child sexual abuse.

## The Present Study

We conducted the current study to see if concerns about the impact of COVID-19 restrictions and the mental health correlates on the risk of online child sexual exploitation or abuse were reflected in self-help seeking by individuals who were concerned about their sexual thoughts or behaviors involving children. To examine this research question, we analyzed service use metrics from two organizations working to prevent child sexual abuse, Stop It Now! USA and the Lucy Faithfull Foundation (operating Stop It Now! UK and Ireland), that provide confidential helplines as well as self-help resources. Specifically, we looked at webpages and helpline contacts that fell under one of three categories: self-help seeking, information for parents/guardians, and information for a general audience. We opted to analyze data from the two organizations to establish whether any changes in webpage/helpline access from one organization would be replicated with data from the sister organization.

We tested the hypothesis that there was an increase in self-help seeking in the first months of the pandemic (i.e., March to July), as many countries entered different versions of lockdown, including the USA and the UK and Ireland. As part of our hypothesis, we also expected to see self-help seeking decrease as time progressed. To see if webpage/helpline traffic changes were unique to self-help seeking we compared self-help seeking to information seeking from parents or guardians of children who were concerned about child safety, as well as general information seeking. As we were simply using information seeking from parents as well as general information seeking as comparison variables, we did not formulate hypotheses related to their increase or decrease of webpage/helpline traffic. We did not have hypotheses regarding how the USA and UK and Ireland metrics would differ. The reason for considering the two organizations was to see if trends found in one country might be found in the other since the USA and UK are both Western countries with similar cultures experiencing the same pandemic (albeit with different regulations and timelines). As the content on the two webpages differed, we were unable to *directly* compare the two countries and instead relied on trends.

## Method

### Stop It Now! USA Webpage Metrics

Stop It Now! USA provided our team with a list of URLs to pages that were divided into three categories based on the content/information provided on each page: self-help, parent/guardian, and general. Self-help pages were those that contained information for those seeking to manage and/or struggling to manage their sexual interests in and/or behaviors with children. Parent/guardian pages were those that contained information for parents concerned about their children’s safety or about their children’s behavior and included questions or concerns about other’s behavior that could impact children’s safety. General pages were those that provided general information about child sexual abuse (e.g., the definition of child sexual abuse), or information about the Stop It Now! USA organization. Categorization of webpages was completed by the first and second author and approved by the team from Stop It Now! USA. Webpage metrics were available from March 2019 (one year prior to the start of the pandemic in March 2020) till January 2022.

There were sometimes multiple URLs to the same page because different links were used for various media campaigns. These URLs were combined and any links that were no longer active were removed. The top ten most viewed pages for each category, using Google Analytics, are listed in Table 1^2^2Beginning in January 2019, two versions of Stop It Now’s USA Google Analytics were erroneously running at the same time, effectively duplicating their page view data. This issue was detected and rectified in July 2020. Because all pages were affected equally, this issue was fixed by halving all page views during this period.. Page views were considered over unique page views because page views count multiple visits from the same IP address. This is important to consider because someone accessing a link multiple times can reflect a greater need for information or help. However, we repeated the analyses described below using unique page views and found no difference in the pattern of results.

### Stop It Now! USA Helpline Inquiries

Stop It Now! USA was able to provide data regarding inquiries to their helplines (phone lines and chat) in two-week increments starting from March 23, 2020, up until January 2022. March 2020 was chosen as the starting point since Stop It Now! UK and Ireland were only able to provide their own helpline data starting from March 2020 (see below). We therefore chose the same starting point for the USA data to increase our ability to compare helpline inquiries between the two organizations. Inquiries were sorted into three categories (self-help, parent, and other/general) based on the reason for contact. Categorization was completed by the Stop It Now! USA team as part of their own data collection and record keeping. The self-help category included people contacting the helplines due to concern about their own thoughts and behaviors; the parent category included people contacting the helplines about a child’s sexual behaviors, concern about someone’s behaviors toward children, or concern about a child’s safety; the other/general category included people calling about CSEM-related questions, professionals calling to ask for advice, and other miscellaneous topics. For our purposes, we only included inquiries that were answered, as missed phone calls could not be categorized. Further, chat lines were closed outside of operating hours, meaning people would not have been able to leave a message.

### Stop It Now! UK and Ireland Webpage Metrics

Stop It Now! UK and Ireland provided us with the monthly page views of their top ten pages across three categories (self-help, parent, and general) from March 2020 to December 2020. This date range was chosen as pandemic restrictions came into force in the UK and Ireland in March 2020 and data were available to us up to December 2020. We developed categories and assigned webpages to the corresponding categories the same way that the USA web metrics were categorized. The top ten most viewed pages per category are available in Table 1. Webpage metrics prior to March 2020 were unavailable because Stop It Now! UK and Ireland had aggregated multiple websites into a single site in February 2020, which prevented comparing page views to 2019 data. While self-help and general pages came from the same website (i.e., https://www.stopitnow.org.uk), the parent webpage data came from their separate website specific to parents and caregivers (i.e., https://www.parentsprotect.co.uk).

Further, one of the general pages (information about Stop It Now! Scotland) was subject to an advertising campaign in partnership with Police Scotland in April and May 2020, which may have contributed to a dramatic spike in page views for those months (i.e., from 564 page views in March 2020, to 46,413 and 35,621 page views in April and May 2020, respectively).

**Table 1:** Stop It Now! USA and UK and Ireland Top 10 Pages for Each Category March 2020

Stop It Now! USA Page Name	Stop It Now! UK and Ireland Page Name
Self-Help	
Worried about your own thoughts and behaviors Help me with my sexual thoughts about my step-daughter Why permission from a child or underage teen doesn’t count What should I do about my sexual thoughts about children? Concerned during diaper changes Can I get arrested for just having thoughts? Wayne’s story of recovery Safety planning for an adult worried about their own sexual thoughts or feelings towards children Edward’s story of treatment What could happen to me if I’ve crossed the line?	Concerned about your own thoughts or behaviours Concerned about use of the internet Help with inappropriate thoughts or behaviour Understanding the behaviour – completed Understanding the behaviour Concerned about use of the internet – get the facts Is it illegal to be a paedophile?^a^ Get the facts Concerned about use of the internet – no grey area Help with inappropriate thoughts or behaviour – self help
Parent/Guardian	
Tip sheet: Warning signs of possible sexual abuse in a child’s behaviors Behavior in children and adolescents What is age-appropriate? How should I deal with my husband’s very mild fondling of my daughters? Comportamientos sexuales adecuados de acuerdo al desarrollo del niño [Age-appropriate child sexual behavior] What should I do after a child tells? How can I tell if my child has been sexually abused? Adults’ behavior toward child My neighbor’s son acted sexually inappropriately with his sister and my son How to recognize concerning behavior between children	Warning signs in children and adults If a child tells you about abuse Police disclosure scheme FAQs Create a family safety plan Internet safety Harmful behaviour in young people and children What is child sexual abuse? Useful links Books to share with children
General	
What might happen after a report is filed? When must a therapist file a report? Crisis hotlines Help and guidance What is child protective services? Prevention tools Defining child sexual abuse When and how to file FAQs on sex offender treatment Warning signs	The Lucy Faithfull Foundation Resources Stop It Now! Scotland^b^ Professionals looking for advice Helpline FAQ How the Stop It Now helpline works Call examples Wales – it’s time we talked about it Have you been abused?

*Note.* One page name is in Spanish as Stop it Now! USA provides information for both English and Spanish speaking people.

^a^While this would normally have been considered a “general” page, this webpage was located under “help with inappropriate thoughts and behaviour” on the Stop It Now! UK and Ireland website. ^b^The Stop It Now! UK and Ireland general page, “Stop It Now! Scotland” was part of a campaign with Scotland Yard in April and May of 2020, potentially impacting the pageviews for those months.

### Stop It Now! UK and Ireland Helpline Inquiries

For Stop It Now! UK and Ireland, we were provided with a total number of callers/emailers as well as percentage breakdowns of the group categories in two-week intervals from March 23, 2020 to November 1, 2020. Initial group categories included: adults concerned about their own behavior; adults concerned about another person’s behavior; professionals; parents and caregivers concerned about a child or young person’s sexual behavior; and adults concerned about a child or young person who was at risk/showing signs of being sexually abused or being groomed online. These categories were further collapsed into three final categories to mirror the categories from the Stop It Now! USA helpline data: Adults concerned about their own behavior became “Self-help,” parents and caregivers concerned about a child became “Parent,” and all other categories became “Other.”

### Data Analyses

#### Webpage Analyses

##### Stop It Now! USA

As we were interested in the effect of the pandemic on page views, compared to the previous year, we decided to look at the top 10 pages for each category, based on page views, over nine time periods: March to June 2019; July to October 2019; November 2019 to February 2020; March to June 2020, July to October 2020; November 2020 to February 2021; March to June 2021; July to October 2021; and November 2021 to January 2022. These time periods were chosen because pandemic restrictions came into force in the United States in March 2020 and data were available to us to January 2022. We first present descriptive statistics (including plotting the data and looking at percent change from the earliest time to the latest time). We then ran a 3 (category) by 9 (time period) ANOVA to look at the effect of page category and time period on page views. We present a post-hoc plot of the top ten self-help URL page views from November 2019 to end of December 2021.

##### Stop It Now! UK and Ireland

As with the USA webpage data, we looked at the top ten pages for each of the three categories, first by running descriptive statistics and plotting the data. As we were only provided with data from March 2020 to December 2020, we conducted a 3 (category) by 10 (month) two-way ANOVA for the top 10 pages from this timespan to see if there was any significant change in page views over time. As mentioned previously, one of the general pages (information on Stop It Now! Scotland) was subject to an advertising campaign in April and May 2020 and therefore experienced a dramatic spike in page views during those two months. We present results both including and excluding this outlier data.

#### Helpline Analyses

For both Stop It Now! USA and Stop It Now! UK and Ireland helpline data, we first ran descriptive statistics (including plotting the data). To examine whether there were significant increases or decreases in people accessing the helpline over time, we conducted a Mann-Kendall non-parametric trend test.

## Results

### Stop It Now! USA Webpage Metrics

**Figure 1:**
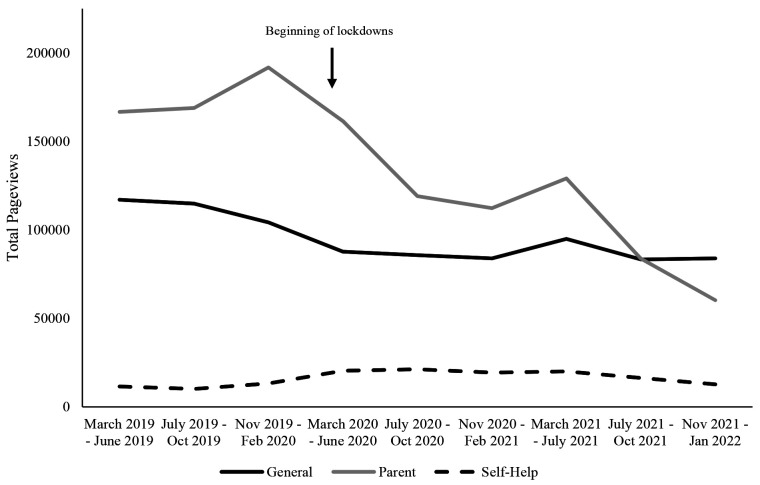
Stop It Now! USA Pageviews for Each Category Across 2020-2022

Figure 1 illustrates category page views over time for the USA website data from March 2019 to January 2022. The figure suggests parent and general page views decreased across much of 2020, with a brief increase from November 2020 – July 2021, followed by a continued decrease.

Self-help page views began to increase around November – February 2020, appearing to stabilize into October 2021, before slightly decreasing again in November 2021 – January 2022. Indeed, when comparing March to June 2019 (the earliest time period) to November 2021 to January 2022 (the latest time period), average parent page views decreased by 64%, average general page views decreased by 28%, and average self-help page views increased by 10%.

Levene’s test of equality of error variances indicated that the assumption of equal variances was violated for pageviews, therefore we report Tukey’s HSD post-hoc results. Tests of between-subjects effects indicated that there was a significant main effect of category on page views, *F*(2, 243) = 45.33, *p* < .001, $\eta_{p}^{2}$ = .27 with parent pages having significantly more views than both general pages and self-help pages. The difference between general page views and self-help page views was also significant, with general pages having higher page views. There was no main effect of time on page views, *F*(8, 23) = 1.35, *p* = .22, $\eta_{p}^{2}$ = .04 nor was there an interaction between category and time on page views, *F*(16, 243) = .83, *p* = .66, $\eta_{p}^{2}$ = .05. Results of both the ANOVA and the post-hoc comparisons of categories are presented in Tables 2 and 3, respectively.

**Table 2:** Descriptives and Results of ANOVA Considering the Effects of Category and Time on Pageviews – USA Web Data

Page Category	*M* (*SD*) Pageviews	ANOVA Results				
		Source	*df*	*F*	*p*	$\eta_{p}^{2}$
Self-Help	1611.65 (2198.04)	Intercept	1	253.34	< .001	.51
Parent	13261.01 (12165.34)	Category	**2**	**45.33**	**< .001**	**.27**
General	9443.89 (7565.50)	Time	8	1.35	.221	.04
		Category x Time	16	0.83	.655	.05

*Note.* Bolded values indicate *p* < .05.

**Table 3:** Post-Hoc Comparisons of Category Page Views – USA Web Data

Page Category	Mean Difference	*p*	95% CI
Parent			
Self-Help	11649.36	< .001	[8707.78, 14590.93]
General	3817.13	.007	[875.55, 6758.70]
General			
Self-Help	7832.23	< .001	[4890.65, 10773.80]

**Figure 2:**
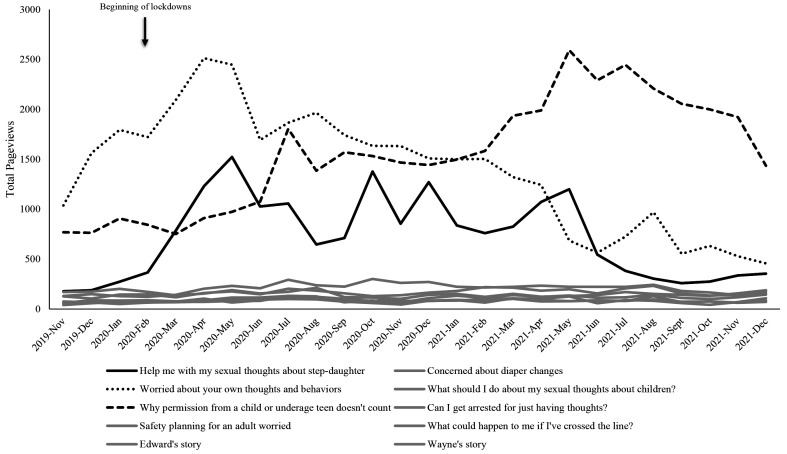
Monthly Page Views for Top 10 Stop It Now! USA Self-Help Pages From November 2019 to December 2021 *Note.* The webpage visits in grey are low and difficult to distinguish because the frequency of visits to these pages did not appear to fluctuate across the pandemic time period. They are included to show the baseline for comparison with the three pages that appeared to show changes over the pandemic.

Because we were interested in how pageviews might have fluctuated for individual self-help pages, we decided to plot pageviews for the top 10 self-help pages post hoc. Figure 2 shows page views for the top 10 self-help pages from November 2019 to the end of December 2021. It is apparent from this visual that three particular self-help pages (worried about your own thoughts and behaviors; why permission from a child or underage teen doesn’t count; and help me with my sexual thoughts about step-daughter) were visited more often than the other seven self-help pages.

### Stop It Now! USA Helpline Inquiries

**Figure 3:**
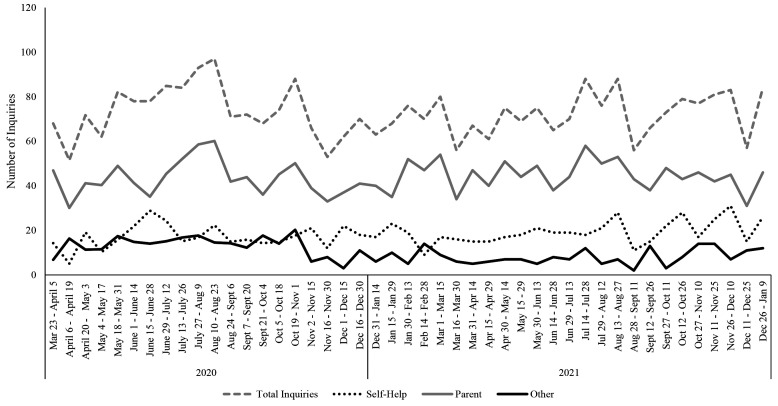
Inquiries (Calls or Chat Messages) to Stop It Now! USA From March 2020 to January 2022 by Category *Note.* Self-help inquiries were those of adults concerned about their own behavior; Parent inquiries were those from parents or caregivers concerned about a child at risk or about a child’s sexual behavior; Other inquiries were those from professionals, questions about CSEM, and “other” categories.

According to the Stop It Now! USA team, across 2020 and 2021, there were low^3^3That the helpline activity was “low” was the subjective perspective of the Stop It Now! USA team. levels of helpline activity overall (1224 and 1803 calls, respectively). However, there was an overall trend of increased activity in August 2020, with a decline in the early fall (see Figure 3). This trend appeared to be driven by an increase in parent inquiries. By contrast, self-help inquiries peaked earlier, in June 2020. General inquiries appeared to remain relatively more stable over time. A Mann-Kendall trend test suggested that there was a significant increase in self-help related calls over time (τ = .23, *p* = .03) as well as a significant decrease in “other” calls over time (τ = -0.29, *p* = .005). There was no significant change for parent-related calls over time (τ = 0.05, *p* = .65).

### Stop It Now! UK and Ireland Webpage Metrics

**Figure 4:**
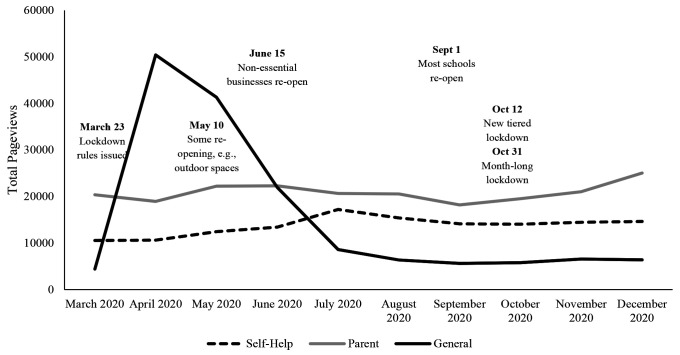
Pageviews From Top 10 Stop It Now! UK and Ireland Webpages From March to December 2020 Across Category *Note.* Outliers included. The peak in general page views in April and May is accounted for by a media campaign that directed web traffic to the Stop It Now Scotland page.

**Figure 5:**
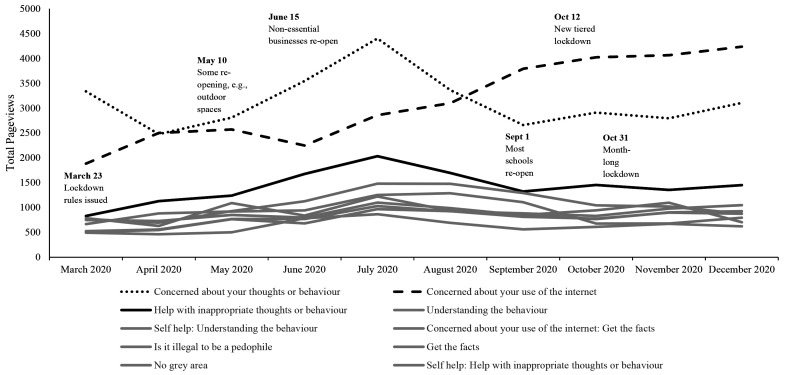
Monthly Page Views for Top 10 Stop It Now! UK and Ireland Self-Help Pages From March to December 2020 *Note.* The webpage visits in grey are low and difficult to distinguish because the frequency of visits to these pages did not appear to fluctuate across the pandemic time period. They are included to show the baseline for comparison with the three pages that appeared to show changes over the pandemic.

Figures 4 and 5 show page view data for the Stop It Now! UK and Ireland webpages. There appeared to be an increase in help-seeking page views during the first months of the pandemic, into July, then a decrease beginning in September. This was true for the top 10 UK self-help pages overall, as well as the top 3 self-help pages (concerned about your own thoughts and behavior; concerned about use of the internet; help with inappropriate thoughts or behavior), paralleling the analysis of Stop It Now! USA pages.

Considering the results with outliers removed, there was a main effect of category on page views, *F*(2, 268) = 12.30, *p* < .001, $\eta_{p}^{2}$ = .08 with the parent pages having significantly more views than self-help pages (*M_diff_* = 718.93, *p* = .02, 95% CI [78.68, 1359.18]) and general pages *(M_diff_* = 1323.25, *p* < .001, 95% CI [679.45, 1967.04]). The difference in page views between self-help and general pages was non-significant. There was no significant main effect of month, nor an interaction between category and month. Results of both the ANOVA and the post-hoc comparisons of categories are presented in Tables 4 and 5, respectively. When we re-ran the analyses including the outlier data, there were no significant main effects or interactions.

**Table 4:** Descriptives and Results of ANOVA Considering the Effects of Category and Time on Pageviews – UK Web Data

Page Category	*M* (*SD*) Pageviews	ANOVA Results				
		Source	*df*	*F*	*p*	$\eta_{p}^{2}$
Self-Help	1369.10 (975.71)	Intercept	1	167.01	< .001	.38
Parent	2088.03 (2434.22)	Category	**2**	**12.30**	**< .001**	**.08**
General	769.39 (1748.83)	Time	9	0.43	.92	.01
		Category x Time	18	0.27	.99	.02

**Table 5:** Post-Hoc Comparisons of Category Page Views – UK Web Data

Page Category	Mean Difference	*p*	95% CI
Parent			
Self-Help	718.93	.02	[92.58, 1345.28]
General	1318.64	< .001	[689.10, 1948.18]
General			
Self-Help	-599.71	.07	[-1229.25, 29.83]

As with the US web data, we opted to plot the top ten self-help pages post-hoc to get a better view of the pattern of webpage views. Figure 5 shows page views for the UK’s top 10 self-help pages from March to December 2020. It is apparent from this visual that three particular self-help pages (concerned about your thoughts or behaviour; concerned about your use of the internet; and help with inappropriate thoughts or behavior) were visited more often than the other seven self-help pages.

### Stop It Now! UK and Ireland Helpline Inquiries

**Figure 6:**
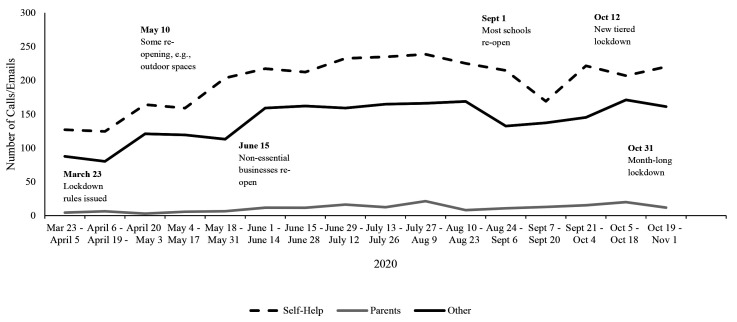
Inquiries (Calls or Emails) to Stop It Now! UK and Ireland From March to November 2020 by Category *Note.* Self-help calls and emails were those of adults concerned about their own behavior; Parent calls and emails were those of parents or carers concerned about a child or young person’s sexual behaviour; Other calls and emails were those from professionals, adults concerned about other adults’ behaviours, and “other” categories.

In examining the data from the Stop It Now! UK and Ireland helpline, all three categories of help-seekers (self-help, parent, and other) seemed to show an increase from the beginning of COVID-related lockdowns to November 2020. Figure 6 offers a visualization of the number of calls and emails received over this timeline, organized by category. A Mann-Kendall trend test suggested that there was a significant increase in self-help related calls over time (τ = .40, *p* = .03) as well as a significant increase in parent calls over time (τ = 0.57, *p* = .003) and “other” calls over time, (τ = 0.53, *p* = .005).

## Discussion

During the COVID-19 lockdowns, police and child protection organizations expressed concerns about an increased risk of child sexual abuse and exploitation due to children spending more time at home, and adults having easier access to children, either in the home or online. For adults struggling with their thoughts and behaviors surrounding children, the increased stress, boredom, and access to children brought on by COVID-19 lockdowns may have resulted in an increase in help-seeking behavior. In the present study, we looked at webpage and helpline metrics from Stop It Now! USA and UK and Ireland both before and during COVID-19 regulations to explore whether there was a change in self-help seeking. We did not have any expectations about how data might differ or look similar between the two countries. While the USA and UK and Ireland are similar in some respects (i.e., Western, educated, industrialized, rich, democratic countries), the also differ in others, such as how they managed their response to the pandemic.

From the descriptive and visual data alone, it appeared that there may have been some support for our hypothesis that self-help seeking had increased since the World Health Organization’s declaration of a COVID-19 pandemic in March 2020. For example, when examining the top 3 self-help pages from Stop It Now! USA (help me with my sexual thoughts about my step-daughter; why permission from a child or underage teen doesn’t count; worried about your own thoughts and behaviors), there appeared to be increases in web-traffic starting around March 2020 and with views continuing to wax and wane as COVID-19 restrictions fluctuated. However, when considering the page view data from both Stop It Now! USA and Stop It Now! UK and Ireland, there was no significant change over time for self-help page views nor for any of the parenting or general pages. It may be that any significant change over time in self-help pages was obscured by low levels of activity, without any change over time in the remaining 7 pages, which pertained to more specific issues such as “concerns about diaper changes” and whether one can get arrested for having sexual thoughts about children. An inverted-U trend was clearer in the UK page view data, with views of self-help pages peaking in July 2020, particularly for the self-help page, “concerned about your thoughts or behaviour”.

One interesting finding from this study was that one of the top 3 viewed webpages for self-help seeking in the USA was a page about sexual thoughts regarding a stepfather. Some stepfathers^4^4Fathers or other parental figures may also have viewed this page because there were no other specific pages for fathers or parents specifically. It may also be relevant that this page was an advice column, where a real person had expressed concerns about their sexual thoughts regarding a stepdaughter. may have experienced an increase in concerns about their thoughts in the first months of the pandemic, when both children and parents/other caregivers were more likely to be at home together for much more time due to school and work closures. This concern may have abated in the summer months, when restrictions were eased and there were more opportunities to be outdoors and apart. The page views for the stepdaughter page increased again once school resumed in September, once again plummeting in the summer months of 2021.

In the UK and Ireland data, one of the top 3 self-help pages had to do with use of the internet. Again, we would expect an increase in concerns about being online (e.g., being tempted to seek out CSEM or to sexually solicit minors online) when restrictions are imposed and people were at home and online much more for school, work, and entertainment, and socially isolated.

In contrast to the webpage analyses, there were significant changes in help-line inquiries over time for both the USA and UK and Ireland. While self-help related calls increased over time in the USA, “other” related calls decreased. In the UK and Ireland, calls increased for all three categories: self-help, parental, and “other”, suggesting that self-help seekers were not unique in looking for information at an increased rate. However, there were fewer time-points for the analysis of the UK and Ireland helpline data compared to the USA (16 time points versus 45, respectively), which may have increased the chances of Type I error. Still, consistent with this idea that pandemic restrictions affected help-seeking, an internal report of calls and emails to the Stop It Now! UK & Ireland helpline conducted after we initiated this study found that 11% of calls from April 1, 2020, to September 30, 2020, mentioned COVID-19 had an impact; of these callers, most (79%) were seeking help for concerns about their own sexual thoughts or behaviors towards children (Nardett et al., 2021). These data should be considered in light of other emerging evidence regarding pandemic-related changes in reporting of online offending (as noted by NCMEC and the Canadian Centre for Child Protection regarding CSEM and online sexual solicitations).

Clearly, there is a discrepancy between the webpage and helpline results. Reflecting on the Stop It Now! USA helpline data, there was a significant increase in people calling for self-help. It might be that the individuals who were most concerned about their sexual thoughts or behaviors felt that they would benefit more from speaking to someone directly rather than searching for information online. While the Stop It Now! websites offer a wealth of information, contacting the helpline would have allowed them to ask more pointed questions about their specific situations. They may also have been connected to other services in their geographic area by contacting the helpline directly. In an earlier study of the limits and benefits of the Stop It Now! helpline in the UK, many callers were individuals who had offended in the past and were making use of the helpline to prevent reoffending (Van Horn et al., 2015). In these cases, it would make sense that these individuals – who are at higher risk of offending by virtue of having previously offended – would seek help from professionals who are well-versed in the area of sexual abuse prevention. Unfortunately, with the present data, we did not have access to caller profiles and cannot speak to the *exact* reasons self-help seekers called the helplines beyond looking for information for themselves.

We did not see noticeable changes in parent/caregiver help seeking throughout the pandemic, except for helpline calls to Stop It Now! UK and Ireland (though all three categories saw increased helpline activity). Perhaps parents and caregivers did have more concerns about the online safety of their children as a result of remote schooling and being online much more during lockdown periods, but they were challenged or overwhelmed by the daily demands of supervising their children and working or otherwise managing during the pandemic. It is notable in this context that Grant et al.’s (2019) earlier analysis of calls to the Stop It Now! USA helpline found the majority of calls were from family members, friends, or concerned bystanders.

### Limitations

We were only able to analyze data from two organizations providing self-help webpages and confidential helplines. It is possible that different results would be obtained from other self-help resources, such as those provided in a language other than English (see Moore Center, 2020 for a non-exhaustive list of non-English self-help resources).

Further, page views do not directly measure concern about committing sexual offenses, as some page views could be explained by curiosity or viewed for other reasons (e.g., by journalists conducting research for a story on perpetration prevention or students researching pandemic resources). To partially control for overall page viewing activity levels, we compared views for self-help pages to views for parent and general pages. In addition, we had data on phone calls and chatlines where the specific queries confirmed it was not simply curiosity.

The nature of these data does not allow us to draw a strong connection to the pandemic, because there may be other reasons for changes (or lack of changes) in webpage views and inquiries. For example, the UK embarked on a major media campaign that dramatically increased views of general information pages and could have increased self-help webpage views and calls as well. Further, we were unable to determine from which countries people were accessing these websites from. Although we can assume that most visitors would be from the USA or UK and Ireland (since a lot of the information was location specific) we are unable to test this assumption.

### Future Directions

Understanding the needs of self-help seekers during COVID-19 restrictions is important for equipping prevention programs to be able to better assist people struggling with their sexual thoughts and behaviors involving children in similar situations in the future. Despite the limitations, we believe that this is a valuable first step in examining the impact of the pandemic on self-help-seeking. Of particular interest would be examining page views across multiple self-help sites, in different languages, as well as logs of IP addresses to see if fluctuations in demand for self-help can be linked to regional fluctuations in pandemic restrictions, to further test the idea that it is pandemic restrictions that are relevant. For example, in the United States, pandemic-related restrictions varied widely by state, with Democratic-led states (e.g., California) generally having stricter lockdowns and closures compared to Republican-led states (e.g., Texas). Comparing self-help activities between states may provide further information about whether restrictions were related to help-seeking behavior. Conversely, Australia and New Zealand had effectively contained COVID transmission with very few to no active cases in the October 2020. We would therefore expect no change in self-help activity from IP addresses geolocated to Australia and New Zealand, or for calls to any self-help resources offered in those countries (if they were available).

Self-help-seeking is only one indicator of potential changes in risk of child sexual exploitation and abuse. Parallel analyses of law enforcement data (arrests, charges, convictions), police intelligence data, reports to child protection agencies, and demand for mental health and social services would also shed light, though all of these sources are constrained by underreporting, which is likely to be exacerbated at this time because of the restrictions and disruptions to child protection services and reporting agencies. To elaborate on an earlier example, children who are being maltreated at home might trigger police and child protection responses if they reported what was happening to a teacher at school, but this is much less likely and more difficult if the child interacts with a teacher with reduced frequency or only in a virtual group learning environment. These different sources of official data would be complemented by self-report surveys of sexual perpetration and victimization involving children.

A basic barrier to all help seeking - self or otherwise - is awareness. Indeed, in a survey of 115 helpline users by Van Horn et al. (2015), lack of awareness of the resources was cited by respondents as a major barrier. About half of the helpline users in the UK, Ireland, and the Netherlands in this analysis by Van Horn et al. were self-help seeking and a quarter were parents or caregivers. Recent research suggests the powerful stigma attached to sexual attraction to children is also a major barrier to help seeking (see Jahnke, 2018). This is an important gap because at least some people who are sexually attracted to children may require support not to act on that attraction. This is well-established in the sexual offending research literature, where sexual attraction to children is an important (though not exclusive) explanation for sexual offending against children among identified perpetrators (see Seto, 2018, 2019). In the community, O’Connor and Gannon (2021) have shown that self-reported sexual interest in children is strongly correlated with propensity to engage in sexual behavior involving children in both university and non-university men. Future research is needed to develop and evaluate interventions to reduce barriers to help seeking. This might include stigma reduction efforts and education campaigns to increase awareness of the available resources.

Another big question is how self-help information and interventions translate to outcomes. Van Horn et al. (2015) suggested information was helpful to help-seekers, but ideally longitudinal follow-up data can be obtained, despite the challenges in collecting such data in terms of user participation, security and privacy protections, and uncertainties about mandatory reporting requirements.

### Conclusion

Our analysis suggests that helplines and self-help websites can play a role in perpetration prevention, especially at times when risk of perpetration might be heightened, such as during a global pandemic. However, barriers such as stigma and lack of awareness of available resources need to be addressed. At the same time, helpline and self-help information are only part of a broad perpetration response, which can include clinician-guided online counseling (see McMahan et al., 2020), self-guided online interventions like Get Help, Troubled Desire and Help Wanted (see Moore Center, 2020), and in-person treatment as in Dunkelfeld (Beier et al., 2009). It would be valuable to see if there were pandemic-related changes in help-seeking for other resources, such as other self-help websites and online interventions, and if there are changes at the local, regional, or national level in response to other crises, such as natural disasters or armed conflicts. Rigorous evaluations of these interventions, as well as confidential helplines and self-help pages, are also needed, in order to identify those that have the most promise in preventing child sexual abuse perpetration.

## Data Availability

Data can be provided upon request to the corresponding author.

## Research Integrity

The authors take responsibility for the integrity of the data, the accuracy of the data analyses, and have made every effort to avoid inflating statistically significant results.

## References

[r1] Australian Institute of Health and Welfare. (2021). *Child protection in the time of COVID-19.* Canberra: AIHW. https://www.aihw.gov.au/reports/child-protection/child-protection-in-the-time-of-covid-19/summary

[r2] Babchishin, K. M., Merdian, H. L., Bartels, R. M., & Perkins, D. (2018). Child sexual exploitation materials offenders. European Psychologist, 23(2), 130–143. 10.1027/1016-9040/a000326

[r3] Beier, K. M., Ahlers, C. J., Goecker, D., Neutze, J., Mundt, I. A., Hupp, E., & Schaefer, G. A. (2009). Can pedophiles be reached for primary prevention of child sexual abuse? First results of the Berlin Prevention Project Dunkelfeld (PPD). Journal of Forensic Psychiatry & Psychology, 20(6), 851–867. 10.1080/14789940903174188

[r4] Ferwana, I., & Varshney, L. R. (2024). The impact of COVID-19 lockdowns on mental health patient populations in the United States. Scientific Reports, 14(1), 5689. 10.1038/s41598-024-55879-9 38454064 PMC10920688

[r5] Grady, M. D., Levenson, J. S., Mesias, G., Kavanagh, S., & Charles, J. (2019). “I can’t talk about that”: Stigma and fear as barriers to preventive services for minor-attracted persons. Stigma and Health, 4(4), 400–410. 10.1037/sah0000154

[r6] Grant, B.-J., Shields, R. T., Tabachnick, J., & Coleman, J. (2019). “I didn’t know where to go”: An examination of Stop It Now!’s sexual abuse prevention helpline. Journal of Interpersonal Violence, 34(20), 4225–4253. 10.1177/0886260519869237

[r7] Hillson, J. M. C., & Kuiper, N. A. (1994). A stress and coping model of child maltreatment. Clinical Psychology Review, 14(4), 261–285. 10.1016/0272-7358(94)90025-6

[r8] INTERPOL. (2020, September 7). INTERPOL report highlights impact of COVID-19 on child sexual abuse. *INTERPOL, News and Events.* https://www.interpol.int/en/News-and-Events/News/2020/INTERPOL-report-highlights-impact-of-COVID-19-on-child-sexual-abuse

[r9] INTERPOL. (2022, May 25). INTERPOL Secretary General: Online child sexual abuse at record levels. *INTERPOL, News and Events.* https://www.interpol.int/en/News-and-Events/News/2022/INTERPOL-Secretary-General-Online-child-sexual-abuse-at-record-levels

[r10] Jahnke, S. (2018). The stigma of pedophilia: Clinical and forensic implications. European Psychologist, 23(2), 144–153. 10.1027/1016-9040/a000325

[r11] McMahan, A., Roche, K., Dreyhaupt, R., Seto, M. C., & Rahm, C. (2024). Changes in sexual thoughts and behaviors in a clinical sample of child sexual abuse material users under the COVID-19 pandemic. Sexual and Relationship Therapy, 39(3), 963–983. 10.1080/14681994.2023.2215710

[r12] McMahan, A., Sparre, C., Söderquist, E., Arver, S., Andersson, G., Kaldo, V., Görts-Öberg, K., & Rahm, C. (2020). Illegal online sexual behavior during the COVID-19 pandemic: A call for action based on experiences from the ongoing Prevent It research study. Archives of Sexual Behavior, 49(5), 1433–1435. 10.1007/s10508-020-01750-7 32488645 PMC7266414

[r13] Moore Center. (2020). *Resources for people concerned about their own sexual thoughts and behavior during the COVID-19 outbreak.* Johns Hopkins Bloomberg School of Public Health. https://www.jhsph.edu/research/centers-and-institutes/moore-center-for-the-prevention-of-child-sexual-abuse/resources/covid-19-csa-prevention

[r14] Nardett, G., Simonet, R., & Findlater, D. (2021). *An initial analysis of the impact of the coronavirus pandemic on callers to the Stop It Now! UK and Ireland helpline*. Lucy Faithfull Foundation.

[r15] National Center for Missing and Exploited Children. (2021, April 30). *COVID-19 and missing and exploited children**.* https://www.missingkids.org/blog/2020/covid-19-and-missing-and-exploited-children

[r16] O’Connor, A., & Gannon, T. A. (2021). An examination of the prevalence and characteristics of UK community males who hold a sexual interest in children using the revised interest in child molestation scale. Psychology, Crime & Law, 27(10), 988–1009. 10.1080/1068316X.2021.1876049

[r17] Owen, G., & Savage, N. (2015, September). The Tor dark net. *Global Commission on Internet Governance* [Paper Series, No. 20]. https://www.cigionline.org/sites/default/files/no20_0.pdf

[r18] Scerri, J., Sammut, A., Cilia Vincenti, S., Grech, P., Galea, M., Scerri, C., Calleja Bitar, D., & Dimech Sant, S. (2021). Reaching out for help: Calls to a mental health helpline prior to and during the COVID-19 pandemic. International Journal of Environmental Research and Public Health, 18(9), 4505. 10.3390/ijerph18094505 33922749 PMC8123060

[r19] Rapoport, E., Reisert, H., Schoeman, E., & Adesman, A. (2021). Reporting of child maltreatment during the SARS-CoV-2 pandemic in New York City from March to May 2020. Child Abuse & Neglect, 116(Pt. 2), 104719. 10.1016/j.chiabu.2020.104719 33162107 PMC7480276

[r20] Schlosser, E., & Estey, C. (2021, January 27). *Online child sexual exploitation trends amid the COVID-19 pandemic* [Online webinar]. Public Safety Webinar Series 2021 – Preventing Online Child Sexual Exploitation, Canada.

[r21] Seto, M. C. (2013). *Internet sex offenders*. American Psychological Association.

[r22] Seto, M. C. (2018). *Pedophilia and sexual offending against children: Theory, assessment, and intervention* (2nd ed.). American Psychological Association. 10.1037/0000107-000

[r23] Seto, M. C. (2019). The motivation-facilitation model of sexual offending. Sexual Abuse, 31(1), 3–24. 10.1177/1079063217720919 28715948

[r24] Sharma, S., Wong, D., Schomberg, J., Knudsen-Robbins, C., Gibbs, D., Berkowitz, C., & Heyming, T. (2021). COVID-19: Differences in sentinel injury and child abuse reporting during a pandemic. Child Abuse & Neglect, 116(Pt. 2), 104990. 10.1016/j.chiabu.2021.104990 33707071 PMC8446928

[r25] Thompson, E. (2020, July 13). Child sex exploitation is on the rise in Canada during the pandemic. *CBC**.* https://www.cbc.ca/news/politics/pandemic-child-sexual-abuse-1.5645315

[r26] Turkington, R., Mulvenna, M., Bond, R., Ennis, E., Potts, C., Moore, C., Hamra, L., Morrissey, J., Isaksen, M., Scowcroft, E., & O’Neill, S. (2020). Behavior of callers to a crisis helpline before and during the COVID-19 pandemic: Quantitative data analysis. JMIR Mental Health, 7(11), e22984. 10.2196/22984 33112759 PMC7652595

[r27] UNICEF. (2020, October). COVID-19 and children. *UNICEF DATA*. https://data.unicef.org/covid-19-and-children/

[r28] Upton, E., Clare, P. J., Aiken, A., Boland, V. C., De Torres, C., Bruno, R., Hutchinson, D., Kypri, K., Mattick, R., McBride, N., & Peacock, A. (2023). Changes in mental health and help-seeking among young Australian adults during the COVID-19 pandemic: A prospective cohort study. Psychological Medicine, 53(3), 687–695. 10.1017/S0033291721001963 33966686 PMC8144825

[r29] Van Horn, J., Eisenberg, M., Nicholls, C. M., Mulder, J., Webster, S., Paskell, C., Brown, A., Stam, J., Kerr, J., & Jago, N. (2015). Stop It Now! A pilot study into the limits and benefits of a free helpline preventing child sexual abuse. Journal of Child Sexual Abuse, 24(8), 853–872. 10.1080/10538712.2015.1088914 26701278

[r30] Yonemoto, N., & Kawashima, Y. (2023). Help-seeking behaviors for mental health problems during the COVID-19 pandemic: A systematic review. Journal of Affective Disorders, 323, 85–100. 10.1016/j.jad.2022.11.043 36435398 PMC9684094

